# Human Blood and Mucosal Regulatory T Cells Express Activation Markers and Inhibitory Receptors in Inflammatory Bowel Disease

**DOI:** 10.1371/journal.pone.0136485

**Published:** 2015-08-25

**Authors:** James D. Lord, Donna M. Shows, Janice Chen, Richard C. Thirlby

**Affiliations:** 1 Translational Research Program at the Benaroya Research Institute at Virginia Mason, Seattle Washington, United States of America; 2 Department of Surgery, Virginia Mason Medical Center, Seattle, Washington, United States of America; Jackson Laboratory, UNITED STATES

## Abstract

**Background:**

FOXP3^+^ regulatory T cells (Tregs) are critical for preventing intestinal inflammation. However, FOXP3^+^ T cells are paradoxically increased in the intestines of patients with the inflammatory bowel disease (IBD) ulcerative colitis (UC) or Crohn’s disease (CD). We determined whether these FOXP3^+^ cells in IBD patients share or lack the phenotype of such cells from patients without IBD.

**Methods:**

We quantified and characterized FOXP3^+^ Treg populations, as well as FOXP3^-^ CD4^+^ T cells, in the lamina propria lymphocytes (LPL) of intestine surgically resected from patients with and without IBD, and in the blood of controls or Crohn’s patients with or without disease activity.

**Results:**

In all samples, a similar fraction of FOXP3^+^ cells expressed the “natural” Treg (nTreg) marker Helios, suggesting that, in IBD, these cells are not entirely “induced” Tregs (iTregs) derived from activated effector T cells. Helios^+^ and Helios^-^ FOXP3^+^ T cells demonstrated similar expression of maturation markers, activation markers, and inhibitory molecules between IBD patients and controls, while FOXP3^-^ cells paradoxically expressed more of the inhibitory receptors CD39, CTLA4, and PD-1 in inflamed mucosa. Greater expression of activation markers was also seen in both Helios^+^ and Helios^-^ Tregs, relative to FOXP3^-^ cells, in both IBD patients and controls, indicating that Tregs are effectively activated by antigen in IBD.

**Conclusions:**

Extensive immunophenotyping revealed that Helios^+^ and Helios^-^ mucosal Tregs exist at a similar frequency, and have a similar expression of inhibitory molecules and activation markers in patients with IBD as in healthy controls.

## Introduction

Although the pathogenesis of IBD remains unclear, a key feature of IBD is dysregulation of the mucosal immune system. CD4^+^ FOXP3^+^ regulatory T cells (Tregs) are a central component of mucosal immune regulation, as humans lacking Tregs due to mutations in the FOXP3 gene develop severe bowel inflammation[1,2]. These Tregs represent a small fraction of total CD4^+^ T cells in blood and tissue, constitutively express CD25 and, upon activation by a cognate antigen, inhibit rather than promote the activity of local immune cells in a contact-dependent manner[3]. While the exact means by which Tregs inhibit an immune response remains obscure, a number of molecular mechanisms have been proposed. Because Tregs constitutively express the high affinity IL-2 receptor, containing CD25[4], they may scavenge the T cell growth factor IL-2 from their environment, thus preventing it from acting on proinflammatory T cells. Many Tregs also express the surface molecule CD39, which is part of a complex involved in degrading the pro-inflammatory molecule ATP[5]. CTLA4 (CD152) is also preferentially expressed by Tregs, and has a powerful role in negative regulation of immune responses through its interactions with B7-1 (CD80) and B7-2 (CD86) on antigen presenting cells (APC)[6]. A similar molecule, PD-1 (CD279), is also involved in immunoregulation through interaction with a pair of receptors, called PD-L1 and PD-L2[7]. More recently, the molecule TIGIT has been found to regulate immune responses through interaction with a pair of receptors, CD112 and CD155[8] in a manner analogous to CTLA4.

Despite a clear loss of immune regulation in the intestines of patients with IBD, the phenotype of human intestinal FOXP3^+^ Tregs remains largely unexplored. Several groups have found no phenotypic defect in the mucosal or mesenteric lymph node (MLN) Tregs of CD or UC patients [9–11]. Paradoxically, a disproportionately high frequency of FOXP3^+^ cells has been described in the lamina propria of diseased bowel from IBD patients[12,13]. Furthermore, we have found that the number of FOXP3^+^ T cells in intestinal biopsies from CD patients correlates positively with the histological grade of inflammation[12]. When isolated from the bowel or draining mesenteric lymph nodes (MLN) of IBD patients, these Tregs have inhibitory activity *in vitro* similar to those isolated from healthy controls[9–11]. However, it is unclear whether *in vitro* suppression assays accurately reflect the *in vivo* activity of Tregs, given the latter’s obvious inability to regulate the mucosal inflammation in IBD. Confounding our ability to study human Tregs is the fact that FOXP3, to date our best marker of Tregs, can also be expressed *de novo* in FOXP3^-^ effector T cells when they are activated in the presence of transforming growth factor beta (TGF-β)[14]. These “induced” Tregs (iTregs) do not express the nuclear factor Helios normally expressed by constitutively FOXP3^+^, thymically derived, “natural” Tregs (nTregs)[15]. Whether or not iTregs share the same inhibitory capacity as nTregs is controversial[16,17]. Thus, it is possible that the plethora of FOXP3^+^ T cells seen in the mucosa of IBD patients are simply activated T cells, without *in vivo* inhibitory activity. Alternatively, because both Tregs and FOXP3^-^effector T cells require activation by cognate antigen in order to function, it is possible that the antigen specificity of Tregs is poorly matched to that of effector T cells in IBD, such that disproportionately more effectors than Tregs are activated by local antigen in the intestinal mucosa.

In some autoimmune diseases, effector T cells may have an intrinsic resistance to the inhibitory effects of otherwise functional Tregs[18,19]. One study reported that effector T cells from the lamina propria of a Crohn’s patient were resistant to suppression by normal donor Tregs *in vitro*[20]. In particular Th17 cells, which are identifiable by their expression of CD161[21,22], have been shown to resist the inhibitory effect of Tregs[23]. We isolated T cells from the homogenized lamina propria lymphocytes (LPL) of IBD patients and controls and found that, in contrast to published data[20], neither CD161^+^ nor CD161^-^ effector T cells from IBD patients were resistant to suppression by a standardized Treg population *in vitro*. We also confirmed that LPL Tregs from these IBD patients have suppressive activity against a standardized effector T cell population. We found that the increased FOXP3^+^ cells described in the mucosa of IBD patients[12,13] were not entirely Helios^-^FOXP3^+^ cells which can arise from activated effector cells. Furthermore, both the Helios^+^ and Helios^-^ FOXP3^+^ cells present in the mucosa of IBD patients were found to express at least as much of the immunoinhibitory receptors CD25, CD39, CTLA4, TIGIT and PD-1 as those of patients without IBD. Finally, we found signs of activation in at least as many FOXP3^+^ cells in the mucosa of IBD patients as in controls. Thus, we have excluded several potential explanations for how Tregs fail to control inflammation in human IBD, while characterizing the phenotypes of Tregs and other CD4^+^ T cells in the human intestinal mucosa.

## Methods

### Subjects

Human subjects research was approved by the institutional review board of the Benaroya Research Institute at Virginia Mason Medical Center. All study subjects (described below) provided written informed consent. Due to the small number of subjects, controlling for environmental and socioeconomic factors was not feasible. All subjects provided consent and all procedures followed IRB-approved protocols. Additionally, as a source of human splenocytes, discarded fresh tissue from the surgically resected healthy spleen of a single patient undergoing distal pancreatectomy for a condition unrelated to cancer or inflammation was obtained with no associated protected health information (PHI), and thus was granted a waiver of consent by the IRB.

Twenty-four consecutive patients (8 with Crohn’s, 7 with UC, 9 without IBD, the latter including 5 with FAP, 3 with colon cancer, 1 with a large adenoma, and 1 with rectal prolapse) undergoing intestinal resection by a single surgeon (RT) were consented to donate portions of resected bowel for research. See Table 1 for surgery patient descriptions. Because IBD patients undergoing surgery are at risk of intraoperative hemorrhage, and are very seldom either asymptomatic or on no medications at the time of surgery, blood was obtained from an independent cohort, consisting of 10 Crohn’s patients (age 26–69, median 47, 22% female) with active symptoms (diarrhea, pain, and/or bleeding), 9 with quiescent disease (age 22–61, median 36, 70% female), and 8 healthy controls (age 22–61, median 34, 63% female). None of the blood donors were on IBD medications at the time of sampling.

**Table 1:** Characteristics of Patients from Whom Intestine Was Evaluated.

	Non-IBD	UC	Crohn's
Age: mean (range), yrs	45 (18–80)	43 (20–70)	40 (25–67)
% male	33%	43%	25%
IBD duration: median (range), yrs	n/a	3 (0.7–10)	12 (4.7–56)
% on steroids	n/a	71%	38%
% on immunomodulators	n/a	14%	50%
% on anti-TNF drugs	n/a	29%	38%
% on 5' ASA drugs	n/a	43%	38%

### Specimen Processing

Where available, inflamed vs. uninflamed intestine (from IBD patients) and ileum vs. colon (from Crohn’s patients) were processed separately with a modified version of published protocols[24]. In short, resected colon mucosa was dissected away from submucosal tissue and stripped of epithelium by serial washes in 37° DTT and EDTA. Residual lamina propria was then chopped and digested in 150 U/mL collagenase-I (Gibco BRL) and 0.1% DNAse (InVitrogen) to liberate individual cells, which were washed in PBS and frozen in DMSO/FCS. Splenocytes were similarly liberated by mechanical dissociation followed by collagenase digestion, and frozen in aliquots. PBMC were harvested by centrifugation over a LymphoPrep (Stemcell Technologies, Vancouver, BC) gradient, and similarly frozen.

### *In vitro* Suppression Assays

Cells described in figure legends were labeled with CFSE (termed “responder” T cells) and cultured with or without sorted CD25^+^, CD127^-^, CD4^+^ Tregs at a 1:2 Treg: responder ratio in the presence of soluble anti-CD3 (OKT3, Janssen-Cilag) for 4 days. Cultures were stained for CD4, CD8, and, in some cases, intracellularly for FOXP3, to evaluate proliferation as the percent of cells in a gated population that diluted CFSE below the lowest mean fluorescence intensity (MFI) of unstimulated cells. In assays where purified CD4^+^ T cells were used in isolation as the responder population, CD3-depleted (via incubation with OKT3 and complement) splenocytes were included in culture to present anti-CD3 and stimulate proliferation.

### Flow Cytometry

Lamina propria lymphocytes (LPL) or PBMC were thawed and stained with a viability dye, followed by extracellular staining with fluorescent antibody panels shown in S1 Table. Cells were then fixed and permeabilized with a kit (eBioscience) for intracellular staining with antibodies to FOXP3, Helios, Ki67, and/or CTLA4, shown in bold on S1 Table. Cells were run on an LSRII flow cytometer and analyzed with FlowJo software.

### Data Analysis

Data was analyzed and graphed with Excel and Prism 5 software. All shown p-values represent two-tailed, nonparametric Mann-Whitney U tests. Given the hypothesis-generating nature of this project, no corrections were made for multiple comparisons. P-values less than 0.05 are shown.

## Results

### CD25^+^/CD127^-^ LPL from IBD mucosa have *in vitro* regulatory function

**Fig 1:**
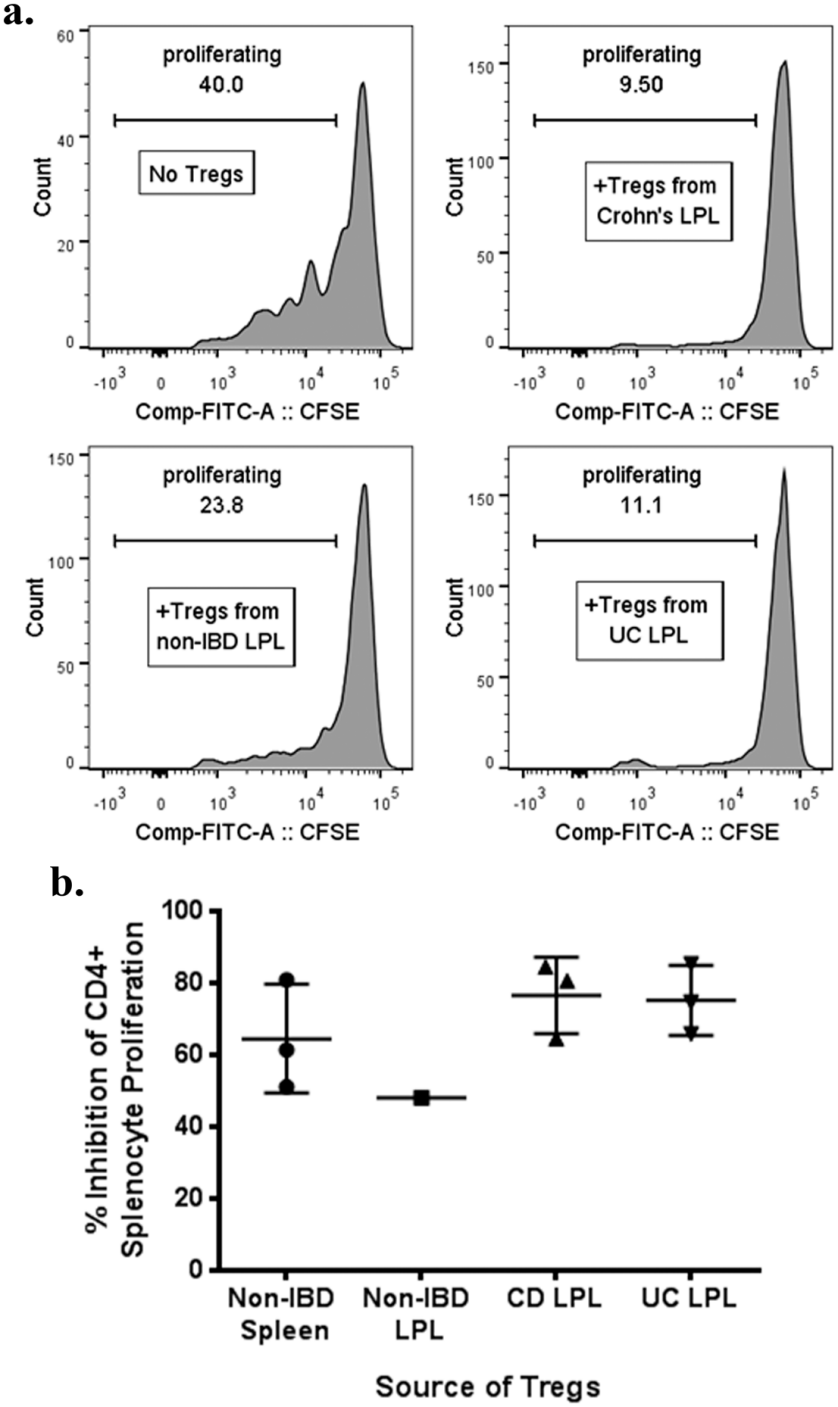
LPL Tregs from IBD patients are suppressive *in vitro*. CD25^+^, CD127^-^, CD4^+^ LPL Tregs homogenized from the surgically resected colons of patients with or without IBD were sorted and added at a 1:2 ratio to CFSE-labeled splenocytes from a single donor without IBD or other inflammatory or malignant conditions. As a control, autologous Tregs were similarly sorted from the splenocytes as well. Cells were cocultured in the presence of soluble anti-CD3 (OKT3) for 4 days and then CFSE dilution by CD4^+^ splenocytes was quantified on a flow cytometer. Representative histograms of CFSE dilution by these CD4^+^ splenocytes in the absence or presence of Tregs from different donors are shown (a). The percent that CD4^+^ splenocyte CFSE dilution was diminished by the presence of LPL Tregs from the indicated donors is shown (b). Each dot is a unique patient, except “Non-IBD Spleen”, for which Tregs from the same spleen donor were isolated *de novo* on each of three different days to serve as a control. CD = Crohn’s disease. UC = ulcerative colitis. LPL = lamina propria lymphocytes.

To confirm previously published data[9] that functional Tregs are present in the intestinal mucosa of IBD patients, we were able to sort sufficient numbers of CD25^+^/CD127^-^ CD4^+^ T cells from the homogenized, surgically resected intestinal LPL of three UC patients, three Crohn’s patients, and one patient without IBD to perform *in vitro* suppression assays. As a consistent source of large numbers of responder T cells for these assays, the splenocytes of a single distal pancreatectomy recipient with no autoimmunity or cancer were used for all experiments. Proliferation of CD4^+^ (Fig 1a and 1b) and CD8^+^ (data not shown) splenocytes was comparably inhibited by the presence of Tregs, regardless of whether the Tregs were from patients with or without IBD.

### Effector T cells from IBD mucosa are sensitive to *in vitro* suppression by Tregs

**Fig 2:**
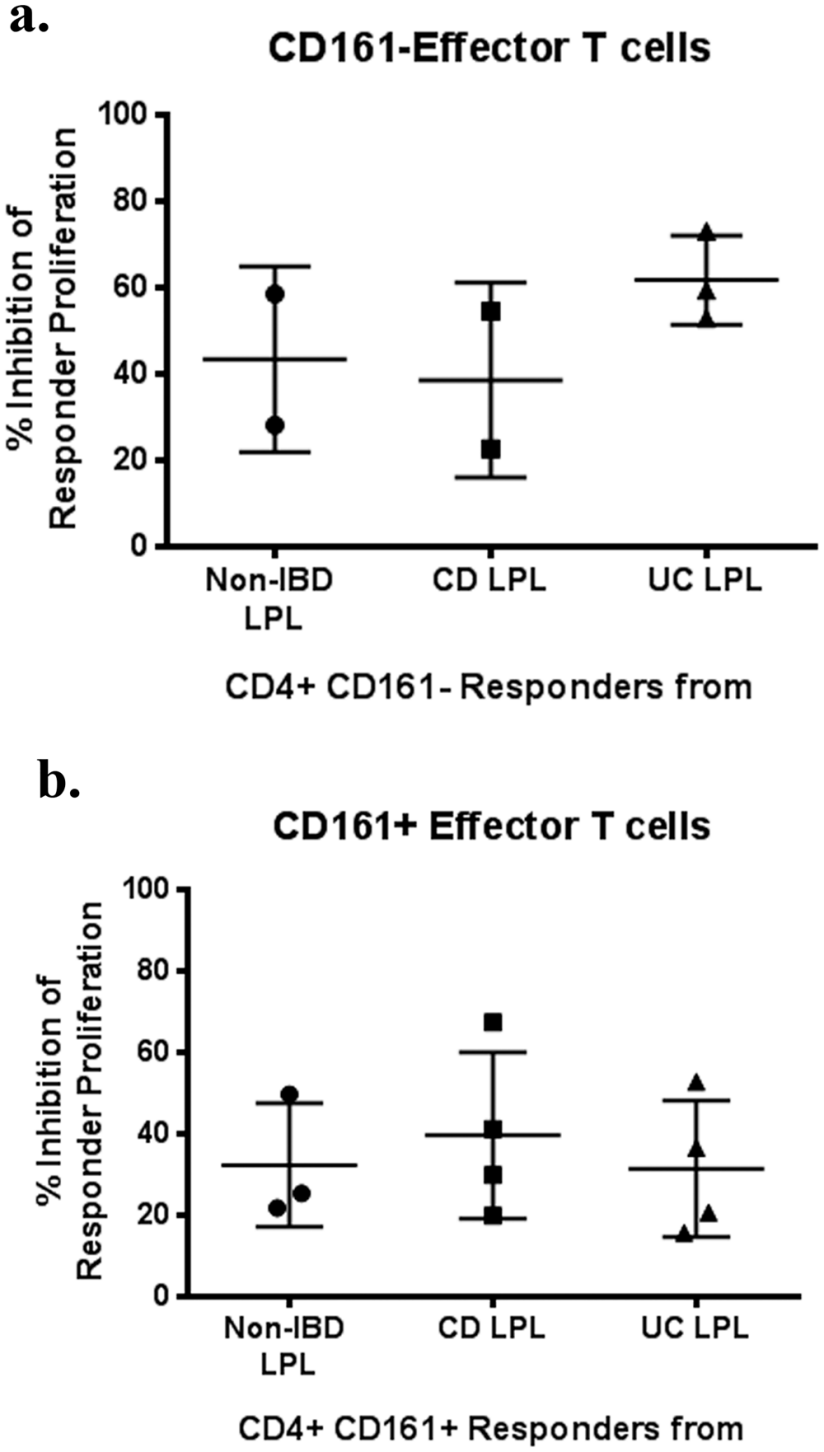
LPL CD4^+^ effector T cells from IBD patients can be suppressed *in vitro* by Tregs. CD4^+^, CD45RA^-^ LPL T cells homogenized from the surgically resected intestines of patients with or without IBD were sorted into CD161^+^ and CD161^-^ subsets, CFSE-labeled, and cultured with or without Tregs sorted from the spleen of a single donor, as in Fig 1, at a 1:2 Treg:effector ratio for 4 days in the presence of soluble anti-CD3 (OKT3). The percent that CFSE dilution by CD4^+^ LPL from the indicated donors was diminished by the presence of splenic Tregs is shown for CD161^-^ (a) and CD161^+^ (b) LPL. CD = Crohn’s disease. UC = ulcerative colitis. LPL = lamina propria lymphocytes.

To determine if in IBD, as in other inflammatory or autoimmune diseases[18,19], effector T cells are insensitive to the suppressive activity of Tregs, we performed the inverse experiment of that described in Fig 1. CD4^+^CD45RA^-^CD25^-^ effector T cells were sorted from LPL of four Crohn’s patients, four UC patients, and three patients without IBD. Because Th17 cells may be enriched in IBD LPL and may be particularly resistant to suppression by Tregs, these CD4^+^ effector T cells were further sorted into populations with or without the Th17 marker CD161. As a consistent source of large numbers of Tregs for these assays, CD4^+^, CD25^+^, CD127^-^ Tregs were harvested from the splenocytes of the aforementioned distal pancreatectomy recipient. LPL effector T cells were less inhibited than splenic effector T cells by the presence of splenic Tregs, particularly if these LPL were CD161^+^ (Fig 2a and 2b). However, the diagnosis of IBD in the effector T cell donor did not further impair Treg responsiveness.

### Helios^+^ nTregs and Helios^-^ iTregs are equally increased in IBD colon

**Fig 3:**
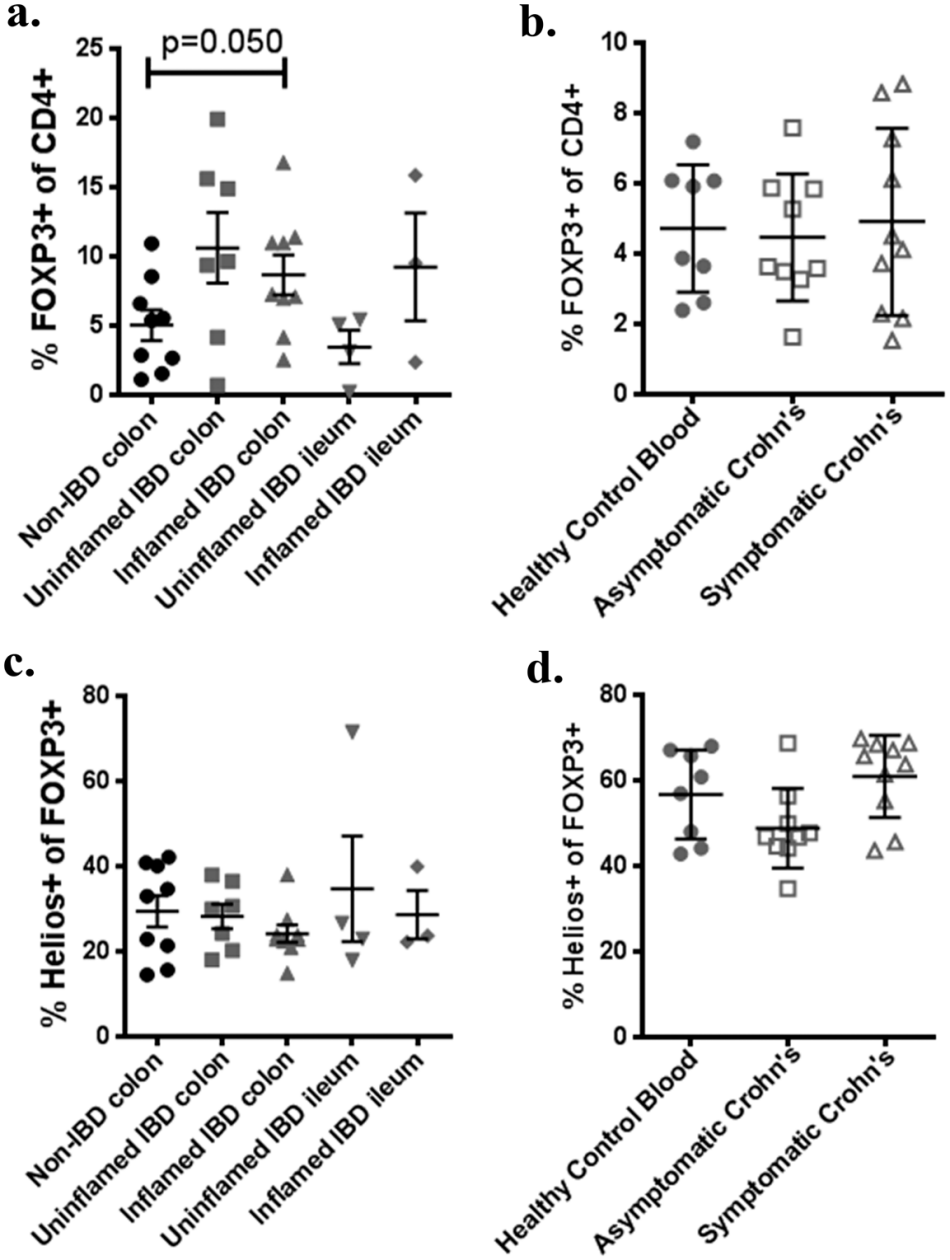
Increased intestinal FOXP3^+^ T cells in IBD are not predominantly Helios^-^. Homegenized LPL (a,c) or PBMC (b,d) were thawed and stained according to Table 1, or with isotype control antibodies. Gating on CD3^+^, CD4^+^ T cells, the percent of colonic or ileal LPL expressing FOXP3 is shown according to whether tissue was inflamed or not (a). The percent of CD3^+^, CD4^+^ PBMC expressing FOXP3 is also shown for a separate cohort of Crohn’s patients, with or without symptoms, or for matched controls (b). Data for a and b was re-gated on CD3^+^, CD4^+^, FOXP3^+^ cells and the percent expressing Helios is shown as above (c, d). Each point represents a unique specimen. Bars indicate means and standard deviations. For c and d, each data point is the mean of three independent assays.

We performed cell-surface and intracellular staining on thawed LPL or PBMC from IBD patients versus controls to identify CD4^+^ FOXP3^+^ Tregs, and divide them into Helios^+^ nTregs and Helios^-^ iTregs. As has been previously described, a greater fraction of the CD4^+^ T cell population was FOXP3^+^ in the colons of IBD patients than controls (p = 0.0307). This trend was seen regardless of whether or not the mucosa from which LPL were derived was grossly inflamed (Fig 3a), but was not seen in the peripheral blood of Crohn’s patients relative to controls, regardless of symptomatic disease activity (Fig 3b). A lower fraction of FOXP3^+^ T cells expressed the nTreg marker Helios in LPL (Fig 3c) than PBMC (Fig 3d) in all cohorts (p<0.0001), but the percent of FOXP3^+^ T cells expressing Helios was no different in the LPL of patients with or without IBD, regardless of inflammation (Fig 3c). Thus the increase in FOXP3+ T cells seen in IBD colon must be similar in both Helios^+^ and Helios^-^ subpopulations. A slightly higher frequency of Tregs expressed Helios in the blood of symptomatic than asymptomatic Crohn’s patients, but not significantly, and neither cohort differed substantially from healthy controls (Fig 3d).

### Surface expression of inhibitory molecules by Tregs is not decreased in IBD

**Fig 4:**
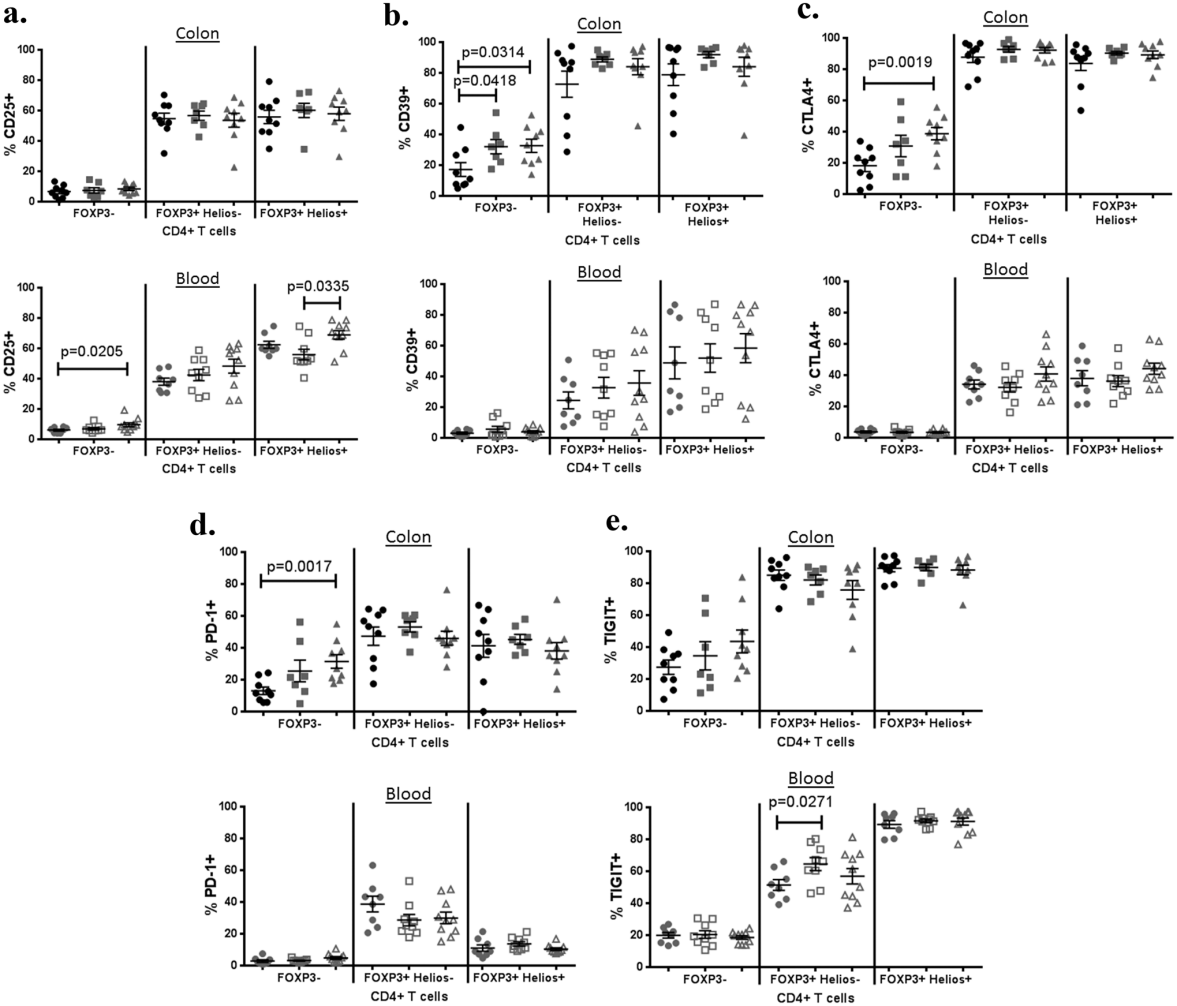
Tregs express inhibitory molecules in IBD no less than in controls. The percent of CD3^+^, CD4^+^ LPL (upper panels) or PBMC (lower panels) expressing CD25 (a), CD39 (b), CTLA4 (c), PD-1 (d), or TIGIT (e) is shown for non-IBD colon (black circles), the uninflamed (gray squares) or inflamed (gray triangles) colon from IBD patients (11 UC, 5 Crohn’s), or for blood from Crohn’s patients with (open gray triangles) or without (open gray squares) active symptoms (diarrhea, pain, and or bleeding), or from age/gender-matched healthy control subjects (gray circles). Positivity is relative to an isotype-matched control antibody for each marker.

Although Tregs isolated from the LPL of IBD patients have been shown to have *in vitro* suppressive activity, some controversy exists as to whether *in vitro* assays of Treg-mediated inhibition accurately reflect their *in vivo* inhibitory activity. Therefore, we instead specifically evaluated the expression of a number of cell-surface receptors which have been associated with Treg inhibitory function, including CD25 (IL-2Rα), CD39 (ENTPD1), CD152 (CTLA4), CD279 (PD-1) and TIGIT (WUCAM, Vstm3) (Fig 4).

In the blood, most of the above molecules were expressed almost exclusively on FOXP3+ T cells, although roughly 20% of FOXP3^-^ cells expressed TIGIT. Among FOXP3+ cells, there was more CD25 (p<0.0001), CD39 (p = 0.0025), and TIGIT (p<0.0001) expression and less PD-1 expression (p<0.0001) in Helios+ nTregs than Helios^-^ iTregs. A modest increase in TIGIT expression by FOXP3^+^, Helios^-^ iTregs was observed in the blood of asymptomatic (but not symptomatic) Crohn’s patients relative to controls. However, there was otherwise no significant difference between the blood of CD patients and controls in the expression of any of these molecules by either Treg subpopulation.

In LPL, there was also no difference between IBD patients and controls in the expression of these immunoinhibitory molecules in either FOXP3^+^ subset of CD4^+^ T cells, as they were expressed by the majority of both iTregs and nTregs in most subjects. Paradoxically, more FOXP3^-^ LPL showed expression of the inhibitory molecules CD39, CTLA4, and PD-1 in IBD patients than controls, and this increase correlated with inflammation. Even in specimens from control subjects, there was greater expression of CD39 (p = 0.0003), CTLA4 (p = 0.0079), and PD-1 (p = 0.0006) in LPL than had been seen in PBMC.

### Mucosal Tregs are activated in IBD

Because both FOXP3^+^ Tregs and FOXP3^-^ effector T cells require activation by antigen to mediate their effects, it is possible that in IBD effectors are being activated to mediate pro-inflammatory activities, while Tregs are not being activated to mediate their anti-inflammatory activities. We therefore evaluated the expression of activation markers on both FOXP3^+^ and ^−^ T cells, further subdividing the former into Helios^+^ and ^−^ Tregs to exclude the possibility that activation markers were exclusively expressed on Helios^-^ iTregs induced from activated effector T cell precursors.

**Fig 5:**
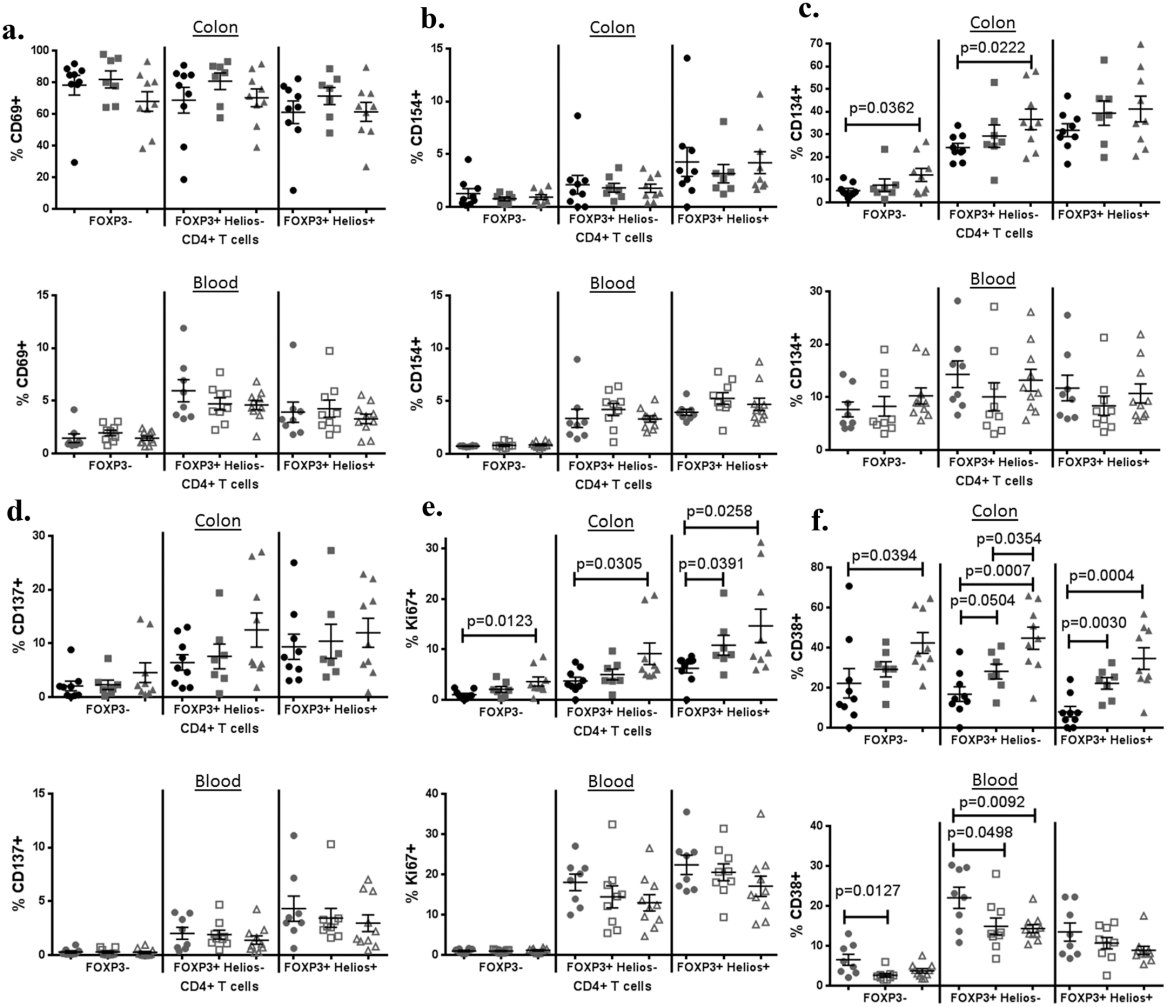
Tregs express activation markers in IBD no less than in controls. The percent of CD3^+^, CD4^+^ LPL (upper panels) or PBMC (lower panels) expressing CD69 (a), CD154 (b), CD134 (c), CD137 (d), Ki67 (e), or CD38 is shown for non-IBD colon (black circles), the uninflamed (gray squares) or inflamed (gray triangles) colon from IBD patients (11 UC, 5 Crohn’s), or for blood from Crohn’s patients with (open gray triangles) or without (open gray squares) active symptoms (diarrhea, pain, and or bleeding), or from age/gender-matched healthy control subjects (gray circles). Positivity is relative to an isotype-matched control antibody for each marker.

In blood, the expression of activation markers CD69, CD154 (CD40L), CD134 (OX40) and CD137 (4-1BB) (Fig 5) was relatively rare, but was nonetheless enriched among FOXP3^+^ cells regardless of whether or not donors had IBD. The proliferation marker Ki67 was also more commonly expressed on both Helios^+^ and Helios^-^ FOXP3^+^ T cells than FOXP3^-^ T cells, as has been described previously[25], but again no significant differences were seen between CD patients and controls, although a trend towards decreased Ki67 expression by FOXP3^+^ populations was observed in symptomatic Crohn’s (Fig 5e). The activation marker CD38 was less commonly expressed in CD patients than controls, particularly within the FOXP3^+^ Helios^-^ (iTreg) subpopulation (Fig 5f). However there was also a significantly lower percentage of FOXP3^-^ cells expressing CD38 in the blood of IBD patients relative to controls, indicating that T cell activation was not biased to the FOXP3^-^ effector compartment in IBD.

In mucosal LPL, a higher T cell expression of certain activation markers was seen than in blood, with a majority of all CD4^+^ T cells expressing CD69 in most subjects, regardless of IBD status (Fig 5a). As in blood, LPL expression of CD154 (Fig 5b), CD134 (Fig 5c), and CD137 (Fig 5d) was higher among FOXP3^+^ than FOXP3^-^ T cells, and this bias was not lost in IBD. However, unlike PBMC, the LPL from inflamed mucosal specimens of IBD patients had significantly higher expression of CD134 in their FOXP3^-^ and FOXP3^+^Helios^-^ T cells than specimens from patients without IBD (Fig 5c). Also unlike PBMC, Ki67^+^ (eg: proliferating) CD4^+^ LPL were more common in inflamed specimens than controls, in both FOXP^+^ and ^−^ subsets (Fig 5e).

CD38 expression in intestinal mucosa differed from its expression in blood in several ways (Fig 5f). In contrast to PBMC, FOXP3^+^ LPL did not express more CD38 than FOXP3^-^ LPL. Furthermore, in both FOXP3^+^ and FOXP3^-^ LPL, a higher percentage of cells expressed CD38 in specimens from IBD patients than in specimens from controls, particularly if the specimens were inflamed.

## Discussion

We performed an extensive immunophenotyping analysis of FOXP3^+^ Tregs and other CD4^+^ T cells in the intestinal lamina propria and blood of individuals with and without IBD, and found no evidence that the mucosal immunodysregulation that typifies IBD is associated with alterations in Treg phenotype. We report that Tregs from IBD patients express ample differentiation, activations, and inhibitory molecules that would support a fully regulatory phenotype. Furthermore, by *in vitro* assays, we confirmed that intestinal Tregs from IBD patients have suppressive activity, and further showed that both CD161^-^ and Th17-containing CD161^+^ effector T cells from the intestines of IBD patients are responsive to suppression by Tregs. Although the latter are somewhat more resistant to inhibition by Tregs, and have been associated with chronic inflammatory conditions, we have previously demonstrated a paradoxically lower frequency of CD161^+^ CD4^+^ T cells in the inflamed than uninflamed colons of IBD patients, with the latter resembling those of uninflamed control patients[26]. Therefore, if anything, the diseased mucosa of IBD patients would be paradoxically enriched with CD161^-^ effector T cells that are more sensitive to Treg-mediated inhibition. While it is possible that effector T cells from the LPL of IBD patients could only have a defective response only to autologous Tregs, not revealed by the allogeneic Tregs used in our study, no such defect was revealed in published reports using auotologous Tregs and effector T cells from the blood[27] or MLN[10,11] of IBD patients relative to controls.

Tregs from IBD patients demonstrated similar Helios expression to subjects without IBD, regardless of whether they were sampled from the blood or intestine. Thus the excess fraction of FOXP3^+^ CD4^+^ T cells we confirmed in the intestinal mucosa of IBD patients does not simply reflect effector T cells transiently up-regulating FOXP3 upon activation in the presence of TGF-β, as such “induced” Tregs do not express Helios. The ability of Helios to differentiate peripherally “induced” from thymically-derived “natural” Tregs has recently been called into question by animal models. However, there is as yet no data to indicate that other markers, such as *FOXP3* promoter methylation, can serve better than Helios to differentiate iTregs from nTregs. Furthermore, whether functional differences exist between these two Treg populations remains controversial. Given their hypothesized ontogeny, one would presume that substantial differences in antigen specificity, and hence TCR repertoire, exist between nTregs and iTregs, resulting in distinct roles for these two populations in the GI tract as have been modeled in mice[28,29]. We have previously shown that the TCR repertoires of Helios^+^ and Helios^-^ Tregs from the human colon show some modest (c. 12–22%) overlap, regardless of IBD, although the TCR repertoire of colonic FOXP3^-^ T cells overlaps minimally (<10%) with Helios^+^ Tregs, even in UC[26], demonstrating that Helios^+^ Tregs are indeed a distinct population from activated effector cells. In our current studies, we do confirm in humans that the majority of FOXP3^+^ T cells in the colon, in contrast to the blood, are Helios^-^. This suggests that most mucosal Tregs are peripherally converted from effector T cells, and thus may have TCR specificity for, and thus inhibit immunoreactivity to, non-self antigens, such as would be encountered in the intestinal lumen.

*In vitro* assays, including ours, have not shown a functional defect in the ability of Tregs from IBD patients to suppress proliferation of effector T cells with polyclonal stimulation[9–11,27], but such assays may not accurately reflect the *in vivo* inhibitory function of Tregs. As *in vivo* assays of Treg function cannot ethically be conducted in humans, and Treg-mediated inhibition is cell-surface contact-dependent, we instead evaluated Treg expression of certain cell-surface molecules through which Tregs have been proposed to mediate inhibition. At the single-cell level, neither the Helios^+^ nor Helios^-^ mucosal Tregs of IBD patients expressed less of the immunoregulatory molecules CD25, CTLA4, CD39, TIGIT or PD-1 than controls. However, Tregs have also been proposed to suppress inflammation by soluble factors, such as IL-10, IL-35, and TGF-β. Although these cytokines were not included in our analyses, we have previously reported no defect in circulating IL-10 or TGF-β levels in the serum of IBD patients relative to controls[30]. However, a more subtle defect, restricted to the intestinal microenvironment or the FOXP3^+^ minority of T cells, would not have been identified in such systemic analyses.

Intestinal T cells generally expressed a more activated phenotype than did cells from the peripheral blood, as would be expected. Among FOXP3^-^ CD4^+^ cells (representing the majority of T cells), CD69 expression was nearly ubiquitous in the colon, while CD38 increased with tissue inflammation. Perhaps as a consequence, activation-induced inhibitory molecules, such as CTLA4, were also paradoxically up-regulated on effector T cells from inflamed colon. FOXP3^+^ Tregs from the intestinal mucosa of IBD patients showed at least as much evidence of activation in the gut as FOXP3^-^ effector T cells, demonstrating that the Tregs were not selectively failing to encounter cognate antigen in IBD. Furthermore, intestinal Tregs from patients with IBD showed a more activated phenotype than Tregs from non-IBD colon, with more of the activation marker CD38 and the proliferation marker Ki67 in IBD, especially when isolated from inflamed colon. This trend is the opposite of what was observed in the blood, where the presence of IBD tended to reduce Ki67 or CD38 expression. Although much of the T cell activation responsible for Ki67 and CD38 up-regulation likely occurs in the intestine itself, the intestinal lamina propria is such a massive lymphoid organ that even a small amount of sequestration of activated T cells from the blood to the intestine could result in their depletion from the peripheral blood. This phenomenon underscores the importance of evaluating primary tissue in IBD, and suggests that peripheral blood data could reflect the opposite of what is actually happening in the intestinal tissues. However, because the blood and intestinal cells investigated in this study were, for practical and ethical reasons, obtained from separate cohorts, it is difficult to draw direct conclusions about their migration based upon these findings.

## Conclusions

Our findings refute the hypotheses that mucosal Tregs fail to control inflammation in IBD because of a defect in the Tregs themselves, or the CD4^+^ effector T cells responding to them. Indeed, the paradoxical increase in Tregs and immunoregulatory molecules we report in the mucosal of IBD patients suggests that the immune system is up-regulating immunoregulatory mechanisms in an abortive reflex to control ongoing inflammation that, for unknown reasons, is able to resist them.

## Supporting Information

S1 Table Listed are the antibody panels used for described flow cytometry, including fluorophors, clones, and manufacturers. (DOCX)
