# Supplementary material for: Human Blood and Mucosal Regulatory T Cells Express Activation Markers and Inhibitory Receptors in Inflammatory Bowel Disease
Source: PLoS One. 2015 Aug 25;10(8):e0136485. doi: 10.1371/journal.pone.0136485 (PMC4548948; doi:10.1371/journal.pone.0136485)
Supplement: S1 Table — (DOCX) [file pone.0136485.s001.docx]

**Supplementary Table 1. Flow Cytometry Antibody Panels**

|  | Panel 1 | | | Panel 2 | | |
| --- | --- | --- | --- | --- | --- | --- |
| Channel | Marker | Ab Clone | Vendor | Marker | Ab Clone | Vendor |
| FITC | CD25 | M-A251 | BD Biosciences | **Ki67** | **B56** | **BD Biosciences** |
| PE | TIGIT | MBSA43 | eBiosciences | OX40 | ACT35 | BD Biosciences |
| APC | **CTLA4** | **BNI3** | **BD Biosciences** | 4-1BB | 4B4-1 | BD Biosciences |
| PerCP/Cy5.5 |  |  |  | PD-1 | eBio J105 | eBiosciences |
| PE/Cy7 | CD39 | eBioA1 | eBiosciences | CD69 | FN50 | eBiosciences |
| APC-eFlour 780 | CD62L | DREG 56 | eBiosciences | CD40L | 24-31 | eBiosciences |
| Pacific Blue | **Helios** | **22F6** | **BioLegend** | **Helios** | **22F6** | **BioLegend** |
| AlexaFlour 700 | **FOXP3** | **PCH101** | **eBiosciences** | **FOXP3** | **PCH101** | **eBiosciences** |
| eFlour 650NC | CD27 | O323 | eBiosciences | CD38 | HB7 | eBiosciences |
| eFlour 605NC | CD45RA | HI100 | eBiosciences | CD45RA | HI100 | eBiosciences |
| V500 | CD4 | RPA-T4 | BD Biosciences | CD4 | RPA-T4 | BD Biosciences |
| PE Texas Red | CD3 | UCHT1 | Beckman Coulter | CD3 | UCHT1 | Beckman Coulter |
